# Geographic variation in the skulls of the horseshoe bats, *Rhinolophus simulator* and *R. cf. simulator*: Determining the relative contributions of adaptation and drift using geometric morphometrics

**DOI:** 10.1002/ece3.8262

**Published:** 2021-11-02

**Authors:** Gregory L. Mutumi, David S. Jacobs, Lunga Bam

**Affiliations:** ^1^ Animal Evolution and Systematics Group (AES) Department of Biological Sciences University of Cape Town Cape Town South Africa; ^2^ Life and Environmental Sciences Department University of California–Merced Merced California USA; ^3^ Radiation Science Department South Africa Nuclear Energy Corporation Pretoria South Africa

**Keywords:** diversification, geometric morphometrics, Lande's model, microevolutionary forces, modularity, neutral evolution, speciation

## Abstract

The relative contributions of adaptation and genetic drift to morphological diversification of the skulls of echolocating mammals were investigated using two horseshoe bat species, *Rhinolophus simulator* and *R. cf. simulator*, as test cases. We used 3D geometric morphometrics to compare the shapes of skulls of the two lineages collected at various localities in southern Africa. Size and shape variation was predominantly attributed to selective forces; the between‐population variance (*B*) was not proportional to the within‐population variance (*W*). Modularity was evident in the crania of *R. simulator* but absent in the crania of *R. cf. simulator* and the mandibles of both species. The skulls of the two lineages thus appeared to be under different selection pressures, despite the overlap in their distributions. Difference in the crania of *R. cf. simulator* was centered largely on the nasal dome region of *R. cf. simulator* but on the cranium and mandibles of *R. simulator*. It is likely that the size and shape of the nasal dome, which acts as a frequency‐dependent acoustic horn, is more crucial in *R. cf. simulator* than in *R. simulator* because of the higher echolocation frequencies used by *R. cf. simulator*. A larger nasal dome in *R. cf. simulator* would allow the emission of higher intensity pulses, resulting in comparable detection distances to that of *R. simulator*. In contrast, selection pressure is probably more pronounced on the mandibles and cranium of *R. simulator* to compensate for the loss in bite force because of its elongated rostrum. The predominance of selection probably reflects the stringent association between environment and the optimal functioning of phenotypic characters associated with echolocation and feeding in bats.

## Graphical abstract

The relative contributions of adaptation and drift to morphological diversification of the crania of echolocating mammals were investigated using two horseshoe bat species, *Rhinolophus simulator* and *R. cf. simulator*, as test cases. Shape variation was predominantly attributed to selective forces. The predominance of selection probably reflects the stringent association between environment and the optimal functioning of phenotypic characters associated with echolocation and feeding in bats.

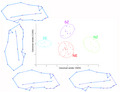

## 1 INTRODUCTION

Understanding the relative contributions of genetic drift and adaptation to organismal diversification is fundamental to studies of evolutionary ecology. To avoid overestimation of selection, genetic drift should always be explicitly accounted for (Betti et al., 2010). However, quantifying the relative contributions of these processes to phenotypic diversification is challenging because distinguishing the two processes and identifying their impacts on diversity are difficult (Brandon, 2005; Brandon & Carson, 1996; Millstein, 2002, 2008). Fortunately, there has been some progress in this regard (Millstein, 2008). Adaptation is deterministic and results in phenotypic patterns correlated with environmental/climatic clines (Millstein, 2008). In contrast, genetic drift is neutral and results from random processes affecting the genetic composition of populations (Millstein, 2008). In many cases, genetic drift is assumed when evidence for selection is not found (Millstein, 2008). However, mathematical approaches, for example, Lande's model (Lande, 1976, 1979) that allow the quantification of the effects of genetic drift on patterns of phenotypic variation, have made it possible to directly determine the relative importance of genetic drift and selection to phenotypic variation. Additionally, assessing modularity can inform the type of selection.

Although the application of Lande's model to phenotypic traits that vary seasonally (e.g., body weight) or are flexible (e.g., behavior) is theoretically possible, for example, Mutumi et al. (2017), application of the model to such data might lead to different results depending on when the traits are sampled. In contrast, hard tissue, for example, bony skeletons including skulls, provides a more permanent record of the evolutionary processes that a species has endured over its history. Several studies have therefore suggested the use of skulls and geometric morphometrics for enquiries into the relative roles of genetic drift and selection, for example, Evin et al. (2008).

Skulls serve functions crucial to the fitness of organisms and their diversification is likely primarily through adaptation (Santana et al., 2012). The neurosensory system (brain), diet acquisition structures, olfactory system, visual system, speech, and sound systems are integrated and housed in the skull. Skulls are therefore subject to diverse selection pressures imposed by the environment on these systems (Cheverud, 1982; Klingenberg, 2008; Pedersen, 1998). For example, the evolution of increased head height, prominent temporal ridge, and huge jaw adductor muscles in Chamaeleonid lizards was associated with strong bite force (Herrel & Holanova, 2008). The association between skull morphology and bite force has also been demonstrated in many other vertebrates (Cleuren et al., 1995; Curtis et al., 2010; Davis et al., 2010; Freeman & Lemen, 2008). For example, elongated snouts in some fish appear to be an adaptation which facilitates feeding through suction (Westneat, 2005). Besides dietary adaptations, other behaviors relevant to fitness have shaped the evolution of skull shape. These are grooming (Rosenberger & Strasser, 1985), fighting with conspecifics (Huyghe et al., 2005), building shelters (Hansell, 2000; Santana & Dumont, 2011; Zuri et al., 1999), and sensing the environment (Oelschläger, 1990; Ross & Kirk, 2007).

The role of genetic drift was demonstrated in the evolution of human skull form and shape (Betti et al., 2010; von Cramon‐Taubadel & Weaver, 2009; Roseman, 2016) using quantitative models. Smith (2011) showed that some parts (basicranium, temporal bone, and face) of the skull evolved neutrally, whereas the mandible evolved through selection. Quantitative and population genetic methods have shown that isolation between Neanderthal and modern human populations led to cranial diversification through genetic drift rather than the commonly proposed adaptive explanations (Weaver et al., 2007). Similarly, Ackermann and Cheverud (2002) and Ackermann and Cheverud (2004) applied Lande's model (Lande, 1976, 1979) to variation in the shape and size of human and monkey skulls and found that genetic drift played a significant role. The role of selection may thus be exaggerated if genetic drift is not accounted for quantitatively (Smith, 2011). This is especially important because studies within the same genus have yielded conflicting results. For example, genetic drift was proposed as the cause of phenotypic convergence and divergence in two horseshoe bats, *Rhinolophus darlingi* (Jacobs et al., 2013) and *Rhinolophus monoceros* (Chen et al., 2009), respectively. In contrast, selection was implicated in the divergence within two other horseshoe bat species, *Rhinolophus capensis* (Odendaal et al., 2014) and *Rhinolophus ferrumequinum* (Sun et al., 2013). Thus, two of the four studies on horseshoe bats (genus Rhinolophus) suggest that selection is the predominant driver of diversification but the other two suggest that genetic drift is the main factor. A rigorous test of the processes behind phenotypic diversification should therefore employ models that weigh the relative contributions of adaptation and genetic drift to determine which is the more dominant process shaping phenotypic variation.

The evolution of skull morphology in animals that rely on acoustic signals for communication or navigation (e.g., bats, dolphins, whales, rodents, and birds) is particularly interesting because it adds a whole suite of selection pressures on the skull besides those associated with diet and the other five senses (Santana & Lofgren, 2013). For example, there are prominent resonant chambers (forming the nasal dome) in the nasal region of the skulls of horseshoe bats (Rhinolophidae), which act as an acoustic horn (Hartley & Suthers, 1988; Pedersen, 1998), allowing echolocation call frequencies to be filtered and emitted at high intensity.

Using 3D geometric morphometrics and Lande's model, we investigated the relative roles of adaptation and genetic drift in two African horseshoe bat lineages, *Rhinolophus simulator* and *R. cf. simulator* (Dool et al., 2016), that are of similar size but differ markedly in the frequency of their echolocation calls. *R. cf. simulator* was previously classified as *Rhinolophus swinnyi*, but genetic analyses, using six nuclear markers and an mtDNA fragment, indicated that individuals originally identified as *R*. *swinnyi* from the northeast of South Africa occurred as a basal lineage to the *simulator* group and most likely represents a cryptic species and sister lineage to *R. simulator* (Dool et al., 2016; see also Taylor et al., 2018). Both nuclear and mitochondrial markers support potential historical or present introgression between *R. simulator* and *R. cf. simulator* since their diversion (Dool et al., 2016; Taylor et al., 2018). We are currently undertaking microsatellite analyses to better understand the structure of the gene pools of *R. simulator* and *R. cf. simulator* and the processes responsible for their genetic similarities despite the marked differences in the frequencies of their echolocation pulses. The frequency of echolocation pulses has a direct impact on the operational range of echolocation and is generally inversely correlated with body size in bats (Jacobs et al., 2007; Jacobs & Bastian, 2018; Jones, 1996, 1999) and with the volume of the nasal dome in the Rhinolophidae (Jacobs et al., 2014). *R. cf. simulator* uses higher‐frequency echolocation calls which are more affected by atmospheric attenuation and probably must emit its calls at greater intensity to achieve the same operational range as *R. simulator*. The skull carries the resonating chambers (the rostral domes) which help to intensify the calls. We therefore hypothesized that selection rather than genetic drift should be the predominant process in the evolution of skull shape because of the vital sensory and foraging functions of the skull. We predicted: (1) significant deviation from proportionality between the within‐ and between‐population trait variance in both species (Ackermann & Cheverud, 2002); and (2) modularity should be more prevalent in the crania of both species than in the mandible because of the central role of echolocation to the survival and reproduction of bats. Independence between the cranium and muzzle allows for relatively more flexible response to sensory‐driven selection. Additionally, the existence of modularity would indicate that the skull is under directional selection because genetic drift and stabilizing selection are inefficient at creating modularity (Melo & Marroig, 2015).

## 2 MATERIALS AND METHODS

### 2.1 Study sites and animals

Skulls were extracted from voucher specimens of both lineages collected in support of two other studies, Mutumi et al. (2016) and Dool et al. (2016). These skulls were supplemented with museum specimens of both lineages (Table A1). A total of 56 crania and 50 mandibles of *R. simulator* and 19 crania and 14 mandibles of *R. cf. simulator* were therefore analyzed. The distributional ranges of the two focal species *R. simulator* (four localities) and *R. cf. simulator* (four localities) follow a latitudinal gradient ranging from 16°S to 32°S in southeastern Africa (figure 1 in Mutumi et al. (2016)). Both *R. simulator* and *R. cf. simulator* lineages have pulses dominated by a constant frequency but at different frequencies with means of 80 and 107 kHz, respectively, when at rest (see fig. S1 in Mutumi et al. (2016)). The two lineages occur in seven woodland types: eastern half of southern Africa, ranging from DRC in the north, through Zimbabwe and Botswana into South Africa in the south. Woodland types include the Central Zambezian miombo woodland in DRC and Zambia, the Zambezian and mopane woodlands, Southern Miombo woodlands, and the Eastern Zimbabwe montane forest–grassland mosaic (Olson et al., 2001). The southern‐most populations occur within Highveld grasslands. In Botswana, the sampling site occurred in an ecotone of three woodlands: Kalahari Acacia–Baekiaea, Kalahari Xeric Savannah, and Southern Africa bushveld. Botswana sites experience the driest climate, and the Eastern Zimbabwe montane forest–grassland mosaic, the wettest (Olson et al., 2001).

**FIGURE A1:**
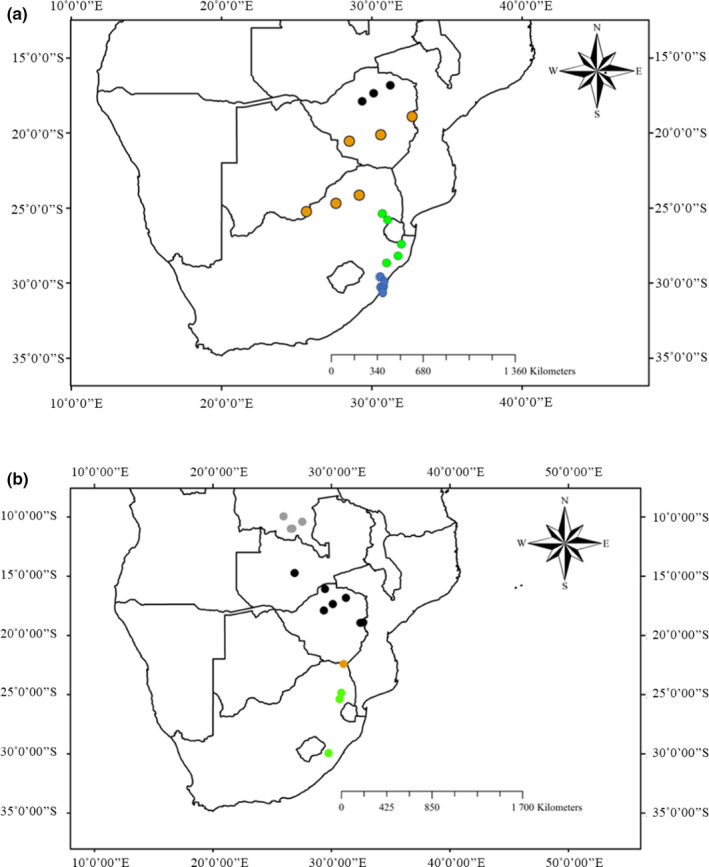
Localities from where skulls and mandibles of (a) *Rhinolophus simulator* and (b) *R. cf. simulator* originated. Groupings used: NZ = northern Zimbabwe (black dots), SZ = southern Zimbabwe and parts of northern South Africa and south of Botswana (orange dots), NE = northeastern South Africa (green dots), SE = southeastern South Africa (blue dots), and DR = Democratic Republic of Congo (grey dots)

The specimens were grouped according to the geographic location where they were captured (Figure A1a,b; Table A1). These locations included northeastern South Africa (NE), northern Zimbabwe and combined southern Zambia (NZ), Democratic Republic of Congo (DR), southeastern South Africa (SE), southern Zimbabwe, and northern South Africa combined (SZ; Figure A1a,b; Table A1).

3D images of each skull were captured through microfocus X‐ray tomography at the South African Nuclear Energy Corporation (NECSA, Pretoria, South Africa; (Hoffman & De Beer, 2012)) following the same procedures as in Jacobs et al. (2014). All images were imported into the 3D imaging software, Avizo (version 8.0; Visualization Sciences Working Group, Merignac, France), as volume files. After creating iso‐surfaces from the volume files in Avizo, files were saved in “Stanford ply” format and opened in Meshlab (version 1.3.3, Visual Computing Lab of ISTI—CNR, Italy) for placing landmarks. Landmarks were chosen depending on their homology (common and repeatable points on all skulls for each lineage). We landmarked a voucher specimen 10 times to calculate the precision of each landmark. Deviation statistics (coefficient of variation—CV) and standard error of the mean (S.E.) were calculated to rank the landmark's precision. Landmarks with the highest coefficient of variation and highest standard error were ranked lowest. We then compared the number of incidents where S.E. was higher than all possible population pairwise differences. To do this, we computed a matrix of population pairwise differences in landmarks to determine which differences were less than the measurement error. All landmarks with more incidents of population pairwise differences lower than S.E.s were discarded. This precision test yielded 24 landmarks for the cranium and 15 for the mandible (Table A2). Landmarks were placed on only the right half of the cranium and the right mandible to control for possible asymmetry (Jacobs et al., 2014). Each landmark in the 3D space had three coordinates (*x*, *y*, and *z*). These sets of three coordinates were used in MorphoJ (version 1.7.0_45; (Klingenberg, 2011)) to analyses shape variation in crania and mandibles of the two lineages across different localities.

Landmark coordinates were analyzed as follows. Firstly, a Procrustes superimposition was done on the coordinates to remove variation because of differences in orientation and scale and to standardize the landmarks in a common coordinate system (Adams et al., 2004). Outliers were checked and extreme cases were double checked against the original volume files. Where necessary, the landmarks were reinserted on the skull images. We first tested the allometric effect of size on shape by regressing centroid sizes against the Procrustes shape coordinates. Where the effect was significant, size‐adjusted residuals of shape were extracted for further analyses. A covariance matrix was generated from the Procrustes coordinates or size adjusted residuals of Procrustes shape coordinates, on which a principal components analysis was performed to explore variation in skull shape among the different localities for each species. A Procrustes ANOVA (provided in MorphoJ software) was used to test the significance of the differences in skull shapes across localities and between sexes. To visualize the shape differences, a canonical variate analysis (CVA) was used. Shape changes in the crania and mandibles were visualized using the wireframe outlines in MorphoJ, which compares shape variations against the average skull shape along each canonical variate (CV) with the outlines at the extremes of each CV. Additionally, we extracted the first shape PC for each species and regressed this against geographic coordinates to further check if there were geographic patterns associated with shape variation. Modularity was also investigated using a priori hypotheses according to Klingenberg (2009). Modularity is the differential evolution of different complexes, each complex consisting of groups of traits that evolve together but relatively autonomously from other such complexes (Cheverud, 1996; Klingenberg, 2005; Wagner, 1996). Processes contributing to modularity can be genetic, developmental, functional, or environmental (Klingenberg, 2005). The mandible was divided into subsets of 5 (ascending ramus—landmarks 1–5) and 10 (alveolar region—landmarks 6–15) landmarks and the cranium was divided into subsets of 11 (basicranium—landmarks 6–16) and 13 (rostrum—landmarks 1–5, and 17–24) landmarks (Table A2) as in Jojić et al. (2015). The strength of association between hypothesized modules and all alternative partitions was tested by the covariance ratio (CR) in R statistics according to Adams and Otárola‐Castillo (2013). The CR measures the strength of association between two blocks, that is, the two modules identified by the covariance matrices of their landmark coordinates compared with the two hypothesized modules (Adams & Otárola‐Castillo, 2013). The CR varies from 0 (completely uncorrelated data) to 1.0 (correlated). The strength of the modularity was also measured by the *Z*_cr_ coefficient which measures the strength of modularity in each structure—the more negative the coefficient, the higher the strength of modularity. Computer simulations have shown that *Z*_cr_ has appropriate statistical properties and reduced levels of misspecification and correctly identifies modular signal, when present (Adams & Collyer, 2019).

### 2.2 Lande's model

The relative contributions of genetic drift and adaptation to the variation in crania and mandible shape/size were tested by applying the principles of Lande's model (Lande, 1976, 1979) in the form of the *β*‐test (Ackermann & Cheverud, 2002), which is described in detail in Mutumi et al. (2017). In summary, the model was developed to account for the relative contributions of drift and adaptation. The model specifies that if an organism has diversified through neutral evolutionary processes (mutation and genetic drift), variation between populations (*B*) of phenotypic characteristics should be directly proportional to the variation within populations (*W*) such that *B* ∝ *W* (Lande, 1979). Significant deviations from this model imply other non‐neutral forces acting on the phenotype of the species, possibly natural selection. The *β*‐test is based on the hypothesis of a log‐linear relationship between the variation in phenotypic characteristics between (*B*) and within (*W*) populations. If the slope of this relationship is not significantly different from 1, the null hypothesis is accepted and the observed variations in phenotypic traits can be attributed to neutral evolutionary processes (mutation and genetic drift). Otherwise, the null hypothesis is rejected, which implies that non‐neutral evolutionary processes, such as natural selection, can be inferred as the dominant driver of diversification.

Successive landmark coordinates were used to generate Euclidean distances (*D*) for successive pairs of landmarks using the following formula:

$$D_{i}=\sqrt{\left({\left(x_{i,1}‐x_{i,2}\right)}^{2}+{\left(y_{i,1}‐y_{i,2}\right)}^{2}+{\left(z_{i,1}‐z_{i,2}\right)}^{2}\right)}$$

where *x*, *y*, and *z* are the 3D landmark coordinates, the subscripts 1 and 2 denote successive positions, and *D_i_* is the Euclidean distance for landmark *i*. This generated a total of 14 inter‐landmark distances for the mandibles, and 23 inter‐landmark distances for the crania.

The resulting multivariate response matrix comprising *D_i_* was used to derive the within‐locality (*W*) and between‐locality (*B*) variances following the procedure outlined in Mutumi et al. (2017). Briefly, the *D_i_* response matrix was fitted using MANOVA with localities and sex as the categorical predictors to generate a variance/covariance (V/CV) matrix for each species. A measure of the within**‐**population variance *W* was then obtained in the form of eigenvalues derived from principal component analysis (PCA) on the V/CV matrix. The between‐population variation *B* was estimated through multiplication of the matrix of PCA‐derived eigenvectors with the matrix of *D_i_* means of each locality. Between‐population variances were calculated by projecting population means on the within‐population PCs to produce new PCs of group mean projections. To do this, the matrix of eigenvectors (obtained from PCA on the V/CV matrix) was multiplied by the matrix of population–phenotype means, a trait (columns) by population (rows) matrix. From the product of the two matrices (eigenvectors and population means), the variances around each product PC factor were calculated. This value represents the between‐group variance. For the regression analysis, only PCs explaining 95% of the variation were used; the rest were discarded as noise. Therefore, the differences in PCs between the two species do not reflect that different landmark points were used for the two species, they were generated from exactly the same landmark coordinates. Additionally, the discarded PCs appeared as extreme outliers. We thought this analysis was appropriate because our aim here was not to detect which parts of phenotype were influenced by genetic drift but rather to detect the overall signal of genetic drift from the whole phenotype. We then regressed the log‐transformed within variance against the log‐transformed between variance and carried out regression t‐tests to test the hypothesis that there was no significant difference between the regression slope and 1 as a function of:

$$\ln\left(B\right)=\beta_{0}+\beta \ln\left(W\right)+\varepsilon$$

where *β*_0_ is the intercept term and *ε* is the error (see Mutumi et al. (2017)).

## 3 RESULTS

Procrustes ANOVA tests did not find significant differences between sexes in both species (both in size and shape of crania and mandibles), except for *R. simulator* mandibles. For *R. simulator*, crania size, *F*_1;54_ = 2.19, *p* = .14; crania shape, *F*_65;3510_ = 1.01, *p* = .46; mandibles size, *F*_1;49_ = 4.77, *p* = .03; and mandible shape *F*_38;1862_ = 2.47, *p* < .0001. For *R. cf. simulator*, crania size, *F*_1;17_ = 1.25, *p* = .28; crania shape, *F*_65;1105_ = 0.93, *p* = .64; mandible size, *F*_1;12_ = 3.97, *p* = .07; and mandible shape, *F*_38;456_ = 0.79, *p* = .81. Sexes were therefore pooled for all analyses, balancing the number of males and females for *R. simulator* mandibles.

The allometric relationship between size and shape was significant for *R. simulator* skulls: crania, *F*_1;56_ = 2.35, *p* = .031; mandibles, *F*_1;50_ = 5.44, *p* = .004. There was no allometric size effect in *R. cf. simulator*: crania, *F*_1;18_ = −0.21, *p* = .507; mandibles, *F*_1;13_ = 1.03, *p* = .155. Therefore, we used size adjusted shape residuals in further analyses of *R. simulator* skulls.

There was variation in the shape of crania across different localities within each lineage (*R. simulator*: *F*_3;56_ = 2.21, *p* = .005; *R. cf. simulator*: *F*_2;16_ = 2.37; *p* < .001) but not in size (*R. simulator* crania: *F*_3;53_ = 0.15, *p* = .93; *R. cf. simulator* crania: *F*_2;16_ = 2.57, *p* = .11). The mandibles of *R. cf. simulator* differed in shape across localities (*F*_2;11_ = 1.52; *p* < .01) but not size (*R. cf. simulator* mandibles: *F*_2;11_ = 1.68; *p* = .23). Those of *R. simulator* were not different in both shape (*F*_3;50_ = 1.70; *p* = .085) and size (*F*_3;47_ = 0.17; *p* = .91). There was no relationship between shape variation and geographic coordinates in both species: *R. simulator* crania Lat ~ PC1: *R* ^2^ = −0.0181; *F*_(1,55)_ = 0.0049; *p* = .9443, Long ~ PC1: *R* ^2^ = −0.0182; *F*_(1,55)_ = 0.0049; *p* = .9876. *R. cf. simulator* crania Lat ~ PC1: *R* ^2^ = −0.0269; *F*_(1,17)_ = 0.5278; *p* = .4774, Long ~ PC1: *R* ^2^ = −0.05533; *F*_(1,17)_ = 0.0049; *p* = .8152. *R. simulator* mandibles Lat ~ PC1: *R* ^2^ = −0.01301; *F*_(1,49)_ = 0.358; *p* = .5524, Long ~ PC1: *R* ^2^ = −0.0185; *F*_(1,49)_ = 0.09015; *p* = .7653. *R. cf. simulator* mandibles Lat ~ PC1: *R* ^2^ = 0.03474; *F*_(1,12)_ = 1.468; *p* = .249, Long ~ PC1: *R* ^2^ = 0.117; *F*_(1,12)_ = 2.723; *p* = .1248.

### 3.1 Crania

**FIGURE 1:**
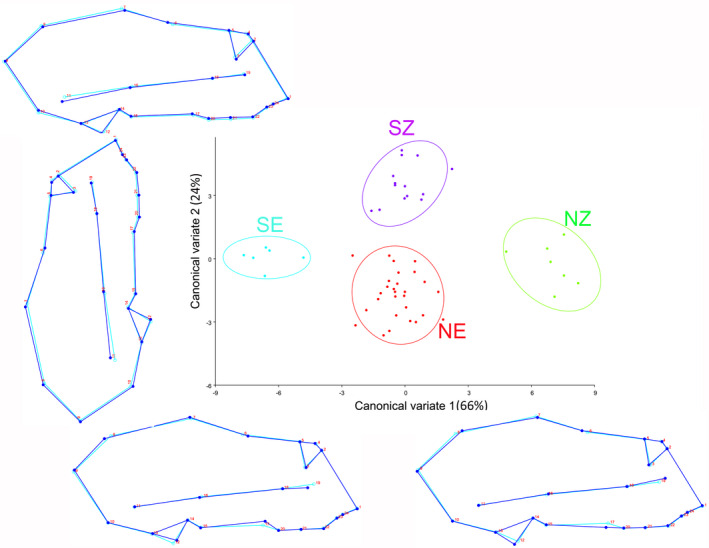
The first two canonical variates of the canonical variate analysis (CV1 and CV2) of crania shape variation among localities of *R. simulator*. Light blue outline represents the average shape; dark blue outline shows the deviation in shape of the cranium from the average. Locality abbreviations: NZ = northern Zimbabwe, SZ = southern Zimbabwe and parts of northern South Africa and south of Botswana, NE = northeastern South Africa, SE = southeastern South Africa, and DR = Democratic Republic of Congo

**FIGURE A2:**
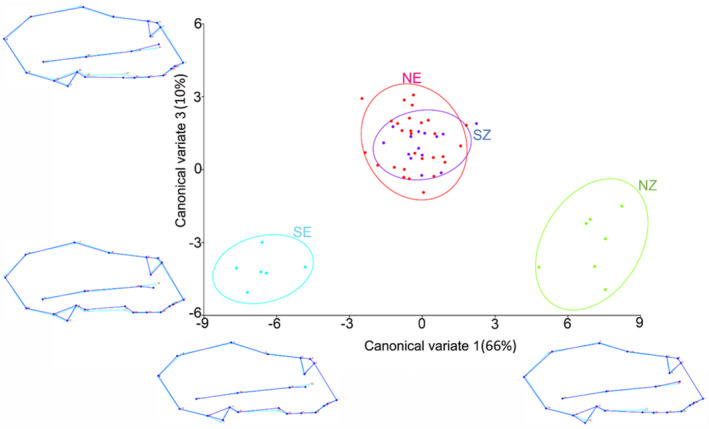
The first and third canonical variates of the canonical variate analysis (CV1 and 3) of skull shape variation among localities of *Rhinolophus simulator*. Light blue outline represents the average shape; Dark blue outline shows the variation in shape of skulls from the average. Locality abbreviations are the same as in Figure 1

For *R. simulator*, the first two canonical variates of the canonical variate analysis (CVA) of shape variation among the localities of *R. simulator* explained a total of 90% of the variation (Figure 1). The wireframe graphs (Figure 1) show that the first canonical variate (CV1) was associated with changes in the palate, zygomatic arch, cranium, and cochlea structure (66% of the variation). Crania from the NZ locality fell at the positive end of CV1 and appeared to have a wider zygomatic arch, broader cochlea, and longer palates relative to the average. Conversely, crania from the SE locality fell at the negative end of CV1 and had a reduced zygomatic arch, a narrower cochlea, and shorter palates relative to the average. Two localities (NE and SZ) fell within the intermediate zone of the CV prescribed shape space, implying that it had a shape close to the average. CV2 was mostly associated with the anterior medial swelling (24% of the variation; Figure 1). The SZ locality fell at the positive end of CV2 and had an outline implying increased volume of the nasal dome relative to the average. Crania from two localities (NE and NZ) fell at the negative end of CV2 indicating that they had a smaller anterior medial swelling than the average, and crania from one locality (SE) were positioned intermediately along CV2 indicating that it had an anterior medial swelling close to the average. CV3 (Figure A2) explained 10% of the variation and was associated with changes in the zygomatic arch and palate. Crania from NZ and SE were on the negative end of CV3, suggesting that they had broader zygomatic arches, and longer palates relative to the average shape and the position of SZ and NE along CV3 indicated that these crania had narrower zygomatic arches and shorter palates relative to the average shape (Figure A2).

**FIGURE 2:**
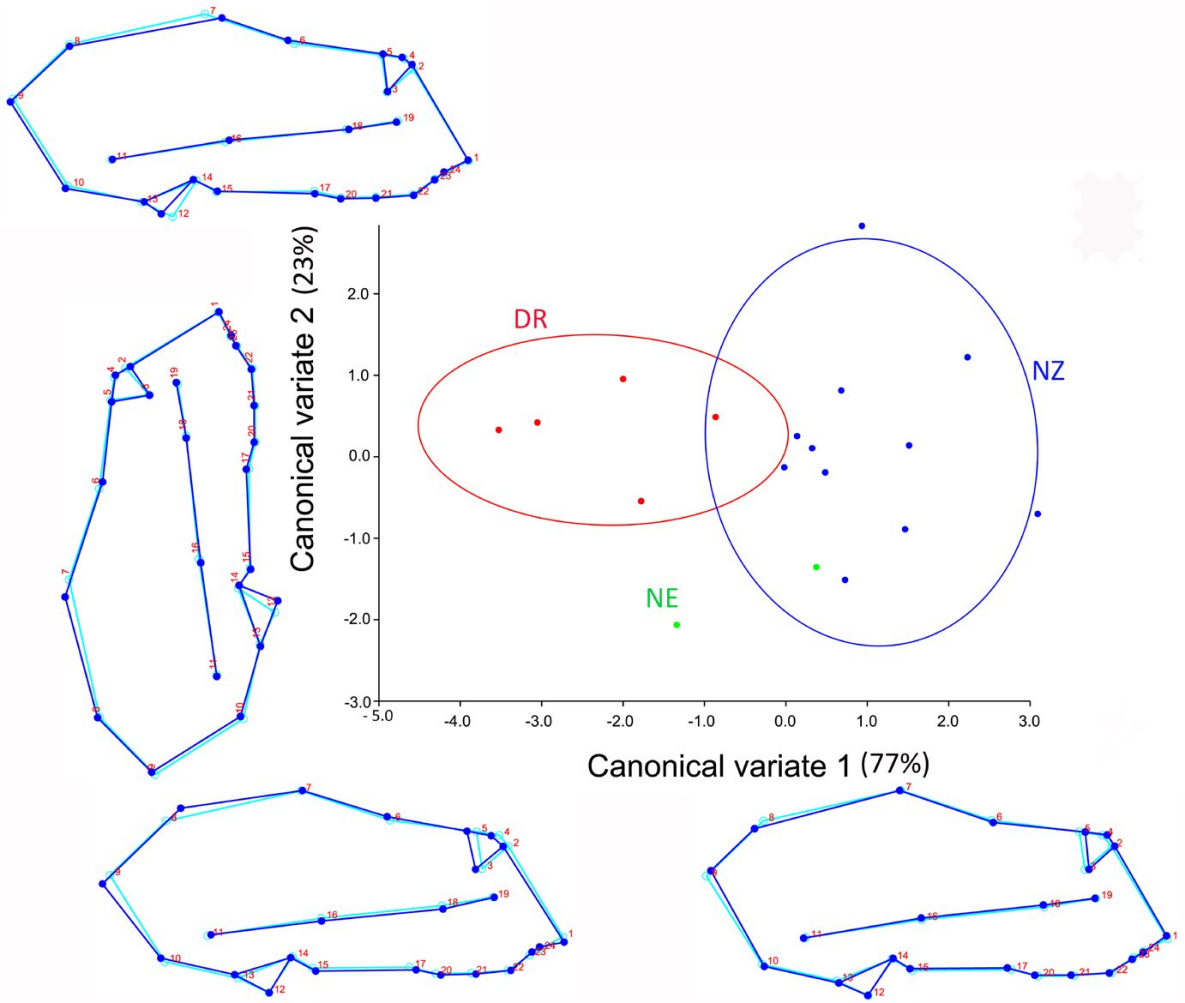
The first two canonical variates of the canonical variate analysis (CV1 and 2) of crania shape variation among localities of *R. cf. simulator*. Light blue outline represents the average shape; Dark blue outline shows the deviation in the shape of crania from the average. Locality abbreviations are the same as in Figure 1

For *R. cf. simulator*, the first two canonical variates of CVA of shape variation among localities explained 100% of the variation (Figure 2). CV1 was associated with changes in the caudal region and anterior medial swelling of the crania, as shown by the wireframe graphs (77% of the variation; Figure 2). The NZ locality fell at the positive end of CV1 and appeared to have a smaller and more anteriorly positioned anterior medial swelling and a narrower and more shortened cranium than the average shape. Conversely, DR locality fell at the negative end of CV1 and had a larger more posterior nasal dome, and a broader and longer cranium relative to the average. One locality (NE) fell within the intermediate shape zone. CV2 was associated with the cochlea and caudal dimensions (Figure 2). All localities seemed to group on the average shape space for CV2 which accounted for 23% of the variation. CV3 could not be derived from the *R. cf. simulator* dataset because of the small sample size.

### 3.2 Mandibles

**FIGURE 3:**
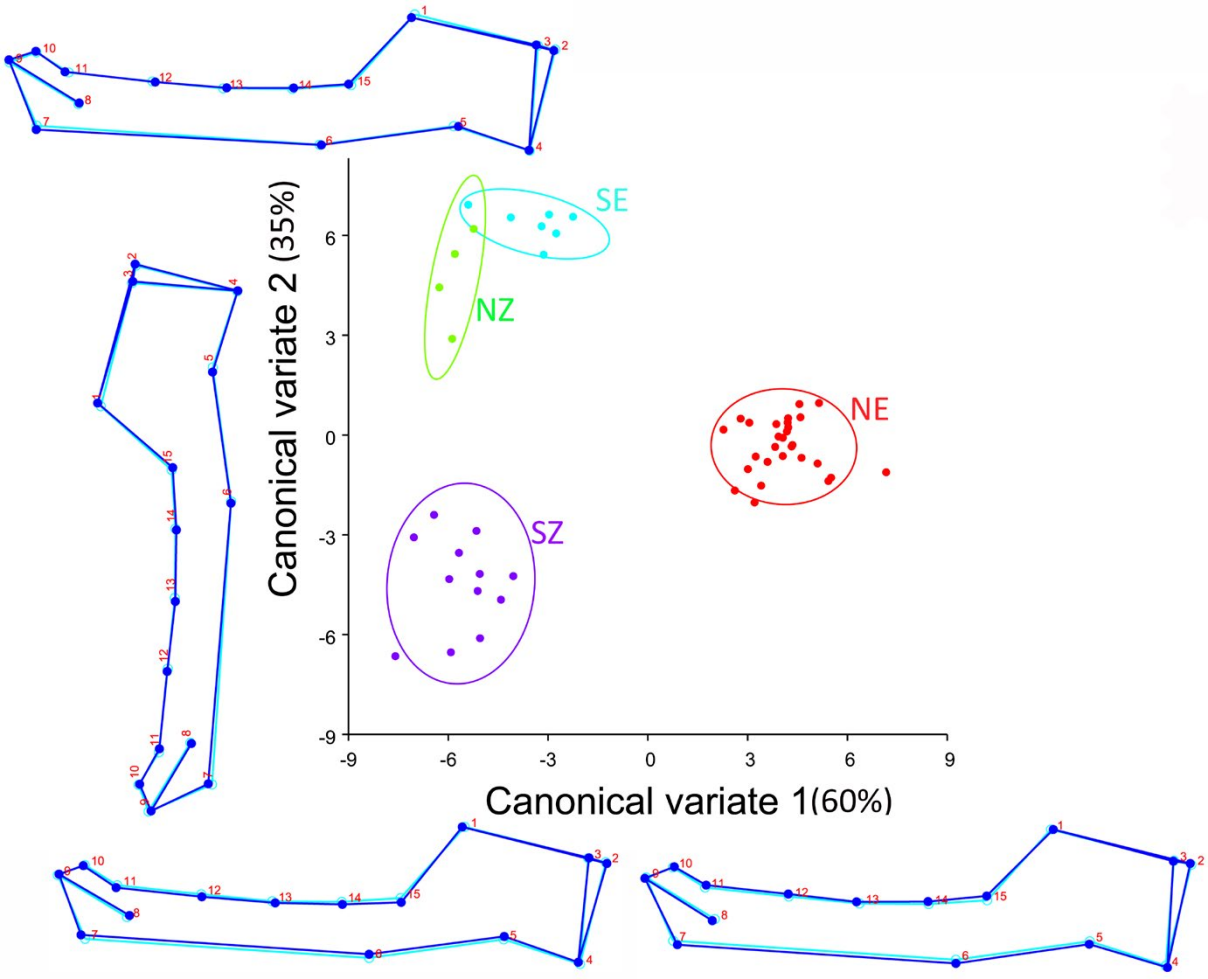
The first two canonical variates of the canonical variate analysis (CV1 and 2) of mandible shape variation among localities of *R. simulator*. Light blue outline represents the average shape; Dark blue outline shows the deviation in the shape of mandibles from the average. Locality abbreviations are the same as in Figure 1

**FIGURE A3:**
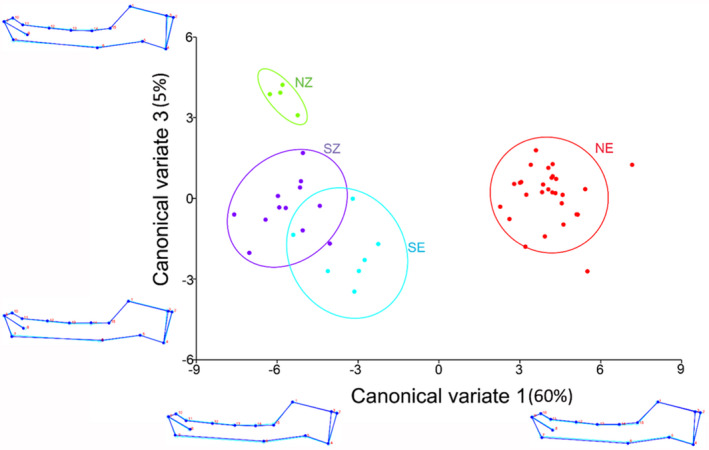
The first and third canonical variates of the canonical variate analysis (CV1 and 3) of mandible shape variation among localities of *Rhinolophus simulator*. Light blue outline represents the average shape; Dark blue outline shows the variation in mandible shapes from the average. Abbreviations are the same as in Figure 1

For *R. simulator*, the first two canonical variates of the canonical variate analysis (CVA) of shape variation among the localities of *R. simulator* explained 95% of the variation (Figure 3). CV1 was only associated with the thickness of the alveolar bone, all the other dimensions seemed consistent with the average shape (60% of the variation; Figure 3). The NE locality fell at the positive end of CV1 and had an outline implying a thicker alveolar bone relative to the average. SZ, NZ, and SE fell at the negative end of CV1 and appeared to have a thinner alveolar bone relative to the average (Figure 3). CV2 was associated with changes in height of the ascending ramus and the thickness of the alveolar bone (35% of the variation; Figure 3). The SE and NZ locality fell at the positive end of CV2 and appeared to have a shorter ascending ramus and a thicker alveolar bone relative to the average. Conversely, SZ locality fell at the negative end of CV2 and had a taller ascending ramus and a thinner alveolar bone relative to the average. NE fell within the intermediate shape zone (Figure 3). CV3 (5% of the variation; Figure A3) did not show much variation in the mandible; all the localities grouped on the average shape space except the NZ locality, which seemed to have a slightly thinner alveolar bone (at the anterior region of the bone).

**FIGURE 4:**
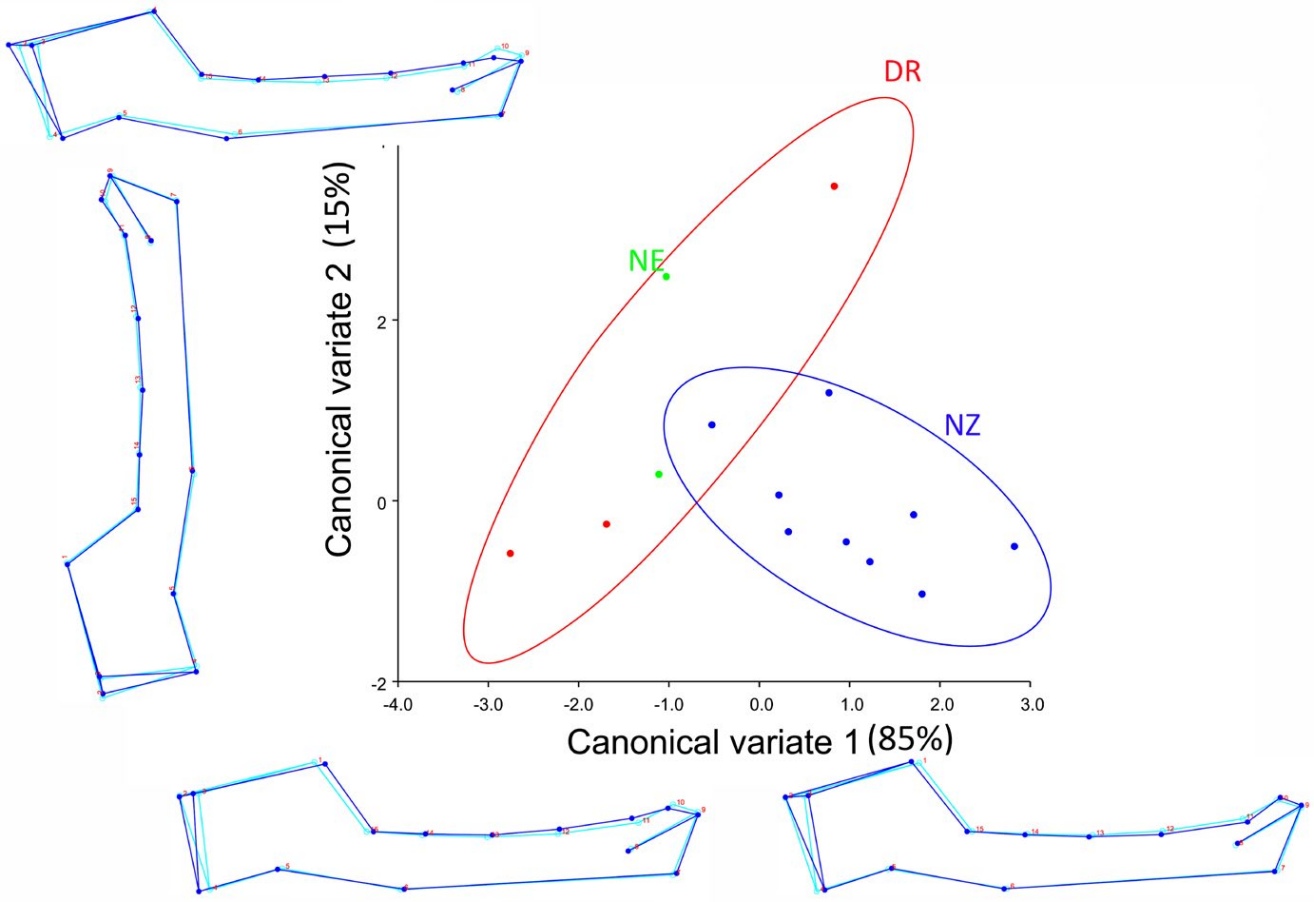
The first two canonical variates of the canonical variate analysis (CV1 and 2) of mandible shape variation among localities of *R. cf. simulator*. Light blue outline represents the average shape; Dark blue outline shows the deviation in the shape of mandible from the average shape. Locality abbreviations are the same as in Figure 1

For *R. cf. simulator*, the first two canonical variates of the CVA of shape variation among the localities explained 100% of the variation (Figure 4). CV1 was associated with changes in the total length of the mandible, thickness of the ascending ramus, and the thickness of the alveolar bone as shown by the wireframe graphs (85% of the variation; Figure 4). The NZ localities fell at the positive end of CV1 and appeared to have a shorter total length of mandible, a thinner ascending ramus, and a thinner alveolar bone than the average shape. Conversely, DR and NE localities fell at the negative end of CV1 and had a longer total length of mandible and thicker alveolar bone relative to the average. CV2 was associated with ascending ramus dimensions and position of the incisor teeth (15% of the variation; Figure 4). The DR locality was at the positive end of CV2 and had an outline implying a shorter ascending ramus and more posterior incisors relative to the average. NZ was at the negative end of CV2, suggesting a slightly longer ascending ramus and slightly posterior incisors than the average shape (Figure 4).

### 3.3 Modularity

The caudal and rostral regions of *R. simulator* crania evolved as separate modules (CR = 0.452, *Z*_cr_ = −2.494 *p* = .004). Contrarily, the mandible of *R. simulator* (ascending ramus and alveolar bone) did not show any modularity (CR = 1.106, *Z*_cr_ = 0.887, *p* = .836). Both crania (CR = 0.821, *Z*_cr_ = −1.276, *p* = .092) and mandibles (CR = 0.859, *Z*_cr_ = −1.168, *p* = .09) of *R. cf. simulator* did not show strong modularity between the partitions analyzed.

### 3.4 Lande's model

**FIGURE A4:**
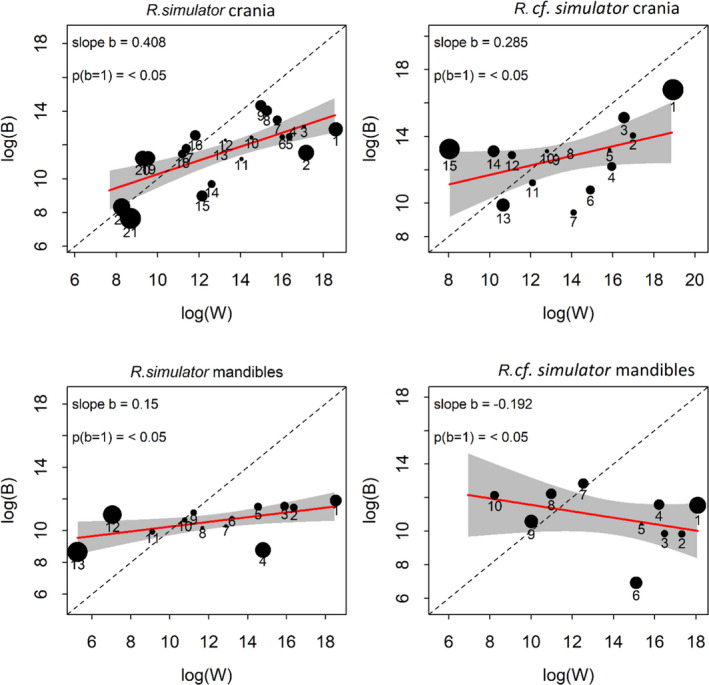
Regression of *B* (between‐group) and *W* (within‐group variance) of skull landmarks in *Rhinolophus simulator* and *R. cf. simulator*. Dot sizes indicate the PC's influence on the regression line (calculated as the difference between the slope values with and without that particular PC point). The regression lines (red lines) are compared to a slope (*b* = 1) based on the null hypothesis of drift (dotted line)

All regression slopes describing the relationship between Ln (*W*) and Ln (*B*) differed significantly from 1 (Table 1) and showed no consistency in the direction of slopes (Figure A4). All the graphs were positive except the *R. cf. simulator* mandibles which showed a negative trend (Figure A4). This indicated that all the tests rejected genetic drift and that the shape and size of crania and mandibles of *R. simulator* and *R. cf. simulator* may have evolved predominantly through selection across different populations. The presence of modularity in the cranium of *R. simulator* suggests that it was under directional selection. The selective pressure responsible for the variation in cranium shape and size in *R. cf. simulator* appeared to be stabilizing because there was no evidence of modularity in the skulls of this species and because the mandibles show the strongest and negative deviation from the slope predicted for genetic drift (Figure A4).

**TABLE 1:** Results from Lande's model on the 3D coordinate landmarks of crania and mandibles of *Rhinolophus simulator* and *R. cf. simulator* from different localities within southern Africa

	*R* ^2^	Slope *b*	Intercept	S.E.	95% Interval (2.5–97.5)	*p* (*β* ≠ 1)	Rejection of drift
*R. simulator* crania	0.492	0.408	6.193	0.093	0.214 to 0.601	*p* < .001	Yes
*R. cf. simulator* crania	0.197	0.285	8.839	0.160	−0.060 to 0.631	*p* < .001	Yes
*R. simulator* mandibles	0.311	0.150	8.756	0.067	0.002 to 0.298	*p* < .001	Yes
*R. cf. simulator* mandibles	0.144	−0.192	13.490	0.165	−0.572 to 0.189	*p* < .001	Yes

Note

Slope *b*: Estimation of regression slope, along with the standard error (S.E.) and *p* (*β* *≠* 1) *p*‐value for the null hypothesis of *b* = 1.

## 4 DISCUSSION

The relationship between the within‐ and between‐group variance did not comply with the predictions of the model for genetic drift. As predicted, geographic variation in the crania and mandibles of both lineages was thus likely the result of selection, in accordance with our first prediction. Modularity was only supported in *R. simulator* crania; the caudal and rostral regions evolved as independent units. Contrary to our second prediction, the mandible of *R. simulator* and both the cranium and mandible of *R. cf. simulator* did not show modularity. Thus, the two closely related lineages (Dool et al., 2016) showed contrasting results with respect to modularity of the crania. The results on modularity (see Melo & Marroig, 2015) suggest that the selection responsible for the diversification of *R. simulator* is predominantly directional (in the crania) and stabilizing in the mandibles, whereas in *R. cf. simulator*, it is mainly stabilizing for both the cranium and the mandible.

Our results contrast with Mutumi et al. (2017), who reported signals for genetic drift in the same species. Additionally, the geographic variation in skulls did not follow any predictable pattern with geography as did echolocation parameters with latitude in Jacobs et al. (2017) and with several environmental parameters in Mutumi et al. (2016). However, these studies were based on a broader range of phenotypic features including flight, size, and echolocation parameters. Perhaps the fact that the skull incorporates several functions (e.g., feeding and echolocation) crucial to fitness causes it to be under severe selection pressure that could eliminate or obscure any genetic drift that might have occurred, and any clear patterns with geography. The head is under the influence of multiple selective pressures because it houses the structures used for a variety of crucial survival and reproduction functions, particularly echolocation. Both lineages appear to have experienced selection pressure associated with echolocation, a key survival trait. Echolocation is a sophisticated sense that varies strongly with the task at hand and environmental conditions (Jacobs et al., 2017; Jakobsen et al., 2013; Luo et al., 2014; Mutumi et al., 2016; Schnitzler et al., 2003).

It is surprising that modularity was present only in *R. simulator* and not in *R. cf. simulator* because modularity has been reported across 22 African and Asian species of rhinolophids (Santana & Lofgren, 2013). This is likely due to the species experiencing different types of selection. Using a quantitative genetics simulation framework, Melo and Marroig (2015) show that between‐module correlations decrease under divergent directional selection thereby promoting modularity. Conversely, stabilizing selection leads to less modularity solely by increasing within‐module correlation because there is no advantage to low between‐module correlations (Melo & Marroig, 2015). The absence of modularity in *R. cf. simulator* may therefore be a consequence of stabilizing selection to retain the adaptive complex among flight, body size, and echolocation. In this respect, the evolution of *R. cf. simulator* is similar to Phyllostomidae, which is tightly integrated and probably evolved under the constraint of preserving adaptive complexes (Hedrick et al., 2020). Body size, wing loading, and echolocation frequency in bats are associated allometrically and are indicative of an adaptive complex (Jacobs et al., 2007; Jacobs & Bastian, 2018; Jones, 1999). With respect to these allometric relationships, *R. cf. simulator* is an average rhinolophid. Its echolocation frequency and wing loading fall within the allometric relationships of the genus (Jacobs et al., 2007; Jacobs & Bastian, 2018).

In contrast to *R. cf. simulator*, there was evidence of modularity in *R. simulator* suggesting that its cranium was under directional selection (Melo & Marroig, 2015). Unlike *R. cf. simulator*, the adaptive complex between echolocation frequency and body size is absent. Although its wing loading scaled allometrically with body size, *R. simulator* echolocated at a lower frequency for its body size (Jacobs et al., 2007; Jacobs & Bastian, 2018). Furthermore, it also had lower echolocation frequencies than would be predicted by the volume of its nasal capsules (Jacobs et al., 2014). This suggests directional selection for lower‐frequency echolocation, possibly to increase the operational range of its echolocation, reflected in the phenotype of the skull associated with echolocation. Lower‐frequency sound undergoes less atmospheric attenuation than high‐frequency sound (Lawrence & Simmons, 1982) and, all else being, the echolocation of *R. simulator* should therefore have longer operational ranges than *R. cf. simulator*, unless it emits echolocation pulse at lower intensities. Currently, the intensities at which these two lineages emit their echolocation pulses are unknown. If the same, the fact that *R. simulator* and *R. cf. simulator* were sometimes caught at the same locality and from the same cave, suggests that their use of different echolocation frequency with consequent differences in the operational range of their echolocation pulses, may be a means of partitioning their foraging habitat, if not their diet. In both lineages, the mandible evolved as one complete module (ascending ramus and alveolar bone) contrary to the mandibular modularity found in *R*. *ferrumequinum* (Jojić et al., 2015). The mandible has therefore possibly evolved under constraint and might be following a line of least evolutionary resistance as in the phyllostomids (Hedrick et al., 2020). The mandible variations across localities did not show any difference between the two species except the variations on the position of the incisors that were seen in *R. cf. simulator* but not in *R. simulator*. The similarities between the mandibles signifying close similarities in diet between the two species.

The marked influence of echolocation on the skull of both *R. simulator* and *R. cf. simulator* is also reflected in variations in the shapes and sizes of cochlea in both species. This suggests that selection has acted strongly on both sound production and perception functions in the two lineages. Variations in the morphology of the cochlea are related to variations in perceptions of sound, particularly in rhinolophids (Davies et al., 2013). For example, in rhinolophids, the cochlear basal turn is expanded, more so than in other bats (Davies et al., 2013), probably because of the well‐developed auditory fovea in this taxon allowing the Doppler shift compensation upon which high duty cycle echolocation is based (Neuweiler, 2003). The frequency of echolocation pulses in rhinolophids is also negatively associated with the length of the basilar membrane length and positively associated with the number of cochlear turns (Davies et al., 2013). These relationships suggest that the cochlea of these bats probably track the acoustic properties of the habitats they occupy, hence the geographic variation reported here in both echolocation frequency and cochlea morphology. The finer details of the mechanistic association between cochlea morphology and echolocation parameters still need to be elucidated using high‐density landmark sampling (Davies et al., 2013).

The differences in the selection pressures experienced by the two lineages are remarkable given the genetic similarity of the two lineages at least in the genetic sequences considered by Dool et al. (2016) and Taylor et al. (2018). The two lineages were indistinguishable across nuclear and mitochondrial sequences used in these two studies. It has been suggested that *R. cf. simulator* is possibly a cryptic lineage, sister to *R. simulator* (Dool et al., 2016), a view supported by the differences in the evolution of skulls reported here. However, the two lineages occur at the same sites and sometimes in the same caves and there is some evidence of introgression between them. This raises the question of how they can maintain such divergent and non‐overlapping echolocation frequencies. The answer to this question requires evolutionary development studies to identify the loci which code for echolocation frequency (e.g., Sun et al. (2020)) and how these loci are assorted during gamete formation.

### 4.1 Limitations

One major limitation of our study is sample size. According to Ackermann (2009), a sample size of 40 per geographic locality would be ideal for drift/adaptation tests using the Lande's model. Currently, there are very limited samples both in the museum and from the field due to the inherent destructive sampling needed to collect skulls. A study with increased sample size will have to wait for the accumulation of skulls, from natural attrition, in museum collections for both species. Small sample sizes make the estimation of covariance for these tests less reliable potentially as a result of singularity of matrices, that is, the number of variables is equal or greater than the number of cases. Matrix singularity increases the chances of false‐positive rates and high rates of model misspecification in detecting modularity. The limited sample size also did not permit analyses by exclusion as in Mutumi et al. (2017). This means that site‐specific signals of genetic drift could not be detected by the current analysis. However, our study nevertheless uncovers the underlying evolutionary processes that have shaped the phenotypes of two little known species. The second limitation pertains to the method of calculating modularity. There are recent suggestions, for example, increasing landmarks and using semi‐landmarks (Cardini, 2019), that could improve modularity calculations. However, we did not entertain these because modularity was not the focus of our study.

## 5 CONCLUSION

Even though the variation in skull shapes and sizes in both *R. cf. simulator* and *R. simulator* is predominantly through selection, the two cryptic species differ in the patterns of geographic variation. Our study highlights that the two species differ in modularity of the cranium; with modularity found in *R. simulator* but not in *R. cf. simulator*. This is despite being sympatric as well as syntopic in parts of their range, with evidence for historic or present introgression between the two lineages (Dool et al., 2016; Taylor et al., 2018). The two lineages thus appear to be responding to different selection pressures within the same habitat. This differential response has resulted in the large difference in their echolocation behavior possibly because of different ecologies resulting from habitat partitioning. Alternatively, habitat partitioning within the same habitats may simply be maintaining differences that evolved in allopatry prior to their syntopic condition. If so, the genes associated with echolocation frequency were probably not involved in the proposed historic or present introgression between these two species.

## CONFLICT OF INTEREST

The authors declare no competing interests.

## AUTHOR CONTRIBUTIONS

**Gregory L. Mutumi:** Conceptualization (equal); Formal analysis (equal); Investigation (equal); Methodology (equal); Project administration (equal); Resources (equal); Software (equal); Supervision (equal); Visualization (equal); Writing‐original draft (equal); Writing‐review & editing (equal). **David S. Jacobs:** Conceptualization (equal); Data curation (equal); Formal analysis (equal); Funding acquisition (lead); Investigation (equal); Methodology (equal); Project administration (lead); Resources (lead); Software (equal); Supervision (lead); Validation (lead); Visualization (equal); Writing‐original draft (equal); Writing‐review & editing (equal). **Lunga Bam:** Data curation (equal); Funding acquisition (equal); Investigation (equal); Project administration (equal); Software (supporting).

### OPEN RESEARCH BADGES

This article has earned an Open Data Badge for making publicly available the digitally‐shareable data necessary to reproduce the reported results. The data is available at https://doi.org/10.6071/M3ZH4F.

## Data Availability

All specimens are deposited in publicly accessible collections (Table A1). Sampling locations, morphological data, and scripts are stored in a publicly accessible repository such as Dryad: DOI https://doi.org/10.6071/M3ZH4F.

## APPENDIX A

**TABLE A1:** Skulls and mandibles of *Rhinolophus simulator* and *Rhinolophus swinnyi* collected from museums and from recent field trips in the Animal Evolution and Systematics Lab, University of Cape Town (AES UCT)

Species	Specimen ID	Museum/Where specimen is stored	Sex	Lat	Long	Group
*Rsi*	130612LRRSI01	AES UCT, Cape Town, South Africa	F	−18.91	32.69	SZ
*Rsi*	190612CHKRSi01	AES UCT, Cape Town, South Africa	F	−17.36	30.13	NZ
*Rsi*	260612MABRSi04	AES UCT, Cape Town, South Africa	M	−17.90	29.37	NZ
*Rsi*	250403UDMGGRSIDSJ1	AES UCT, Cape Town, South Africa	F	−25.38	30.69	NE
*Rsi*	040612MTPRSI03	AES UCT, Cape Town, South Africa	M	−20.55	28.51	SZ
*Rsi*	070612MTPRSI02	AES UCT, Cape Town, South Africa	M	−20.55	28.51	SZ
*Rsi*	200612DAMRS103	AES UCT, Cape Town, South Africa	F	−16.83	31.23	NZ
*Rsi*	200612DAMRS106	AES UCT, Cape Town, South Africa	F	−16.83	31.23	NZ
*Rsi*	100612MUSRSi03	AES UCT, Cape Town, South Africa	F	−20.12	30.60	SZ
*Rsi*	190612CHKRSi03	AES UCT, Cape Town, South Africa	F	−17.36	30.13	NZ
*Rsi*	KM18571	Amathole Museum, King Williams Town, South Africa	F	−29.60	30.52	SE
*Rsi*	KM23691	Amathole Museum, King Williams Town, South Africa	F	−29.60	30.52	SE
*Rsi*	KM23699	Amathole Museum, King Williams Town, South Africa	M	−29.60	30.52	SE
*Rsi*	23684F	Amathole Museum, King Williams Town, South Africa	F	−30.27	30.59	SE
*Rsi*	23685M	Amathole Museum, King Williams Town, South Africa	M	−30.27	30.59	SE
*Rsi*	26088‐F	Amathole Museum, King Williams Town, South Africa	F	−30.27	30.59	SE
*Rsi*	K17_18573	Amathole Museum, King Williams Town, South Africa	F	−28.67	30.98	NE
*Rsi*	KM18572	Amathole Museum, King Williams Town, South Africa	F	−28.67	30.98	NE
*Rsi*	KM23676	Amathole Museum, King Williams Town, South Africa	F	−28.67	30.98	NE
*Rsi*	KM23678	Amathole Museum, King Williams Town, South Africa	F	−28.67	30.98	NE
*Rsi*	KM23679F	Amathole Museum, King Williams Town, South Africa	F	−28.67	30.98	NE
*Rsi*	KM23680	Amathole Museum, King Williams Town, South Africa	F	−28.67	30.98	NE
*Rsi*	KM23681F	Amathole Museum, King Williams Town, South Africa	F	−28.67	30.98	NE
*Rsi*	KM23682	Amathole Museum, King Williams Town, South Africa	M	−28.67	30.98	NE
*Rsi*	KM23683	Amathole Museum, King Williams Town, South Africa	M	−28.67	30.98	NE
*Rsi*	KM23686	Amathole Museum, King Williams Town, South Africa	M	−29.60	30.52	SE
*Rsi*	KM23687F	Amathole Museum, King Williams Town, South Africa	F	−29.60	30.52	SE
*Rsi*	KM23688	Amathole Museum, King Williams Town, South Africa	M	−29.60	30.52	SE
*Rsi*	KM23692M	Amathole Museum, King Williams Town, South Africa	M	−29.60	30.52	SE
*Rsi*	KM23693	Amathole Museum, King Williams Town, South Africa	M	−29.60	30.52	SE
*Rsi*	KM23695	Amathole Museum, King Williams Town, South Africa	F	−29.60	30.52	SE
*Rsi*	KM23696F	Amathole Museum, King Williams Town, South Africa	F	−29.60	30.52	SE
*Rsi*	KM23698	Amathole Museum, King Williams Town, South Africa	F	−29.60	30.52	SE
*Rsi*	KM23702M	Amathole Museum, King Williams Town, South Africa	M	−29.60	30.52	SE
*Rsi*	KM23703	Amathole Museum, King Williams Town, South Africa	F	−29.60	30.52	SE
*Rsi*	KM23706	Amathole Museum, King Williams Town, South Africa	M	−29.60	30.52	SE
*Rsi*	KM23711F	Amathole Museum, King Williams Town, South Africa	F	−29.60	30.60	SE
*Rsi*	KM23712	Amathole Museum, King Williams Town, South Africa	F	−30.27	30.60	SE
*Rsi*	KM23713	Amathole Museum, King Williams Town, South Africa	M	−28.20	31.75	NE
*Rsi*	KM23714	Amathole Museum, King Williams Town, South Africa	M	−28.20	31.75	NE
*Rsi*	23689	Amathole Museum, King Williams Town, South Africa	F	−29.60	30.52	SE
*Rsi*	23694	Amathole Museum, King Williams Town, South Africa	M	−29.60	30.52	SE
*Rsi*	23701	Amathole Museum, King Williams Town, South Africa	M	−29.60	30.52	SE
*Rsi*	23700	Amathole Museum, King Williams Town, South Africa	M	−29.60	30.52	SE
*Rsi*	23705	Amathole Museum, King Williams Town, South Africa	M	−29.60	30.52	SE
*Rsi*	TM29787	Ditsong Museum, Pretoria, South Africa	M	−30.27	30.59	SE
*Rsi*	TM41324	Ditsong Museum, Pretoria, South Africa	M	−25.79	31.05	NE
*Rsi*	TM1652	Ditsong Museum, Pretoria, South Africa	M	−24.69	27.62	SZ
*Rsi*	TM45214	Ditsong Museum, Pretoria, South Africa	F	−24.69	27.62	SZ
*Rsi*	TM45215	Ditsong Museum, Pretoria, South Africa	M	−24.69	27.62	SZ
*Rsi*	TM45217	Ditsong Museum, Pretoria, South Africa	M	−24.69	27.62	SZ
*Rsi*	TM45219	Ditsong Museum, Pretoria, South Africa	F	−24.69	27.62	SZ
*Rsi*	TM45221	Ditsong Museum, Pretoria, South Africa	F	−24.69	27.62	SZ
*Rsi*	DM3562	Durban Natural Science Museum, Durban, South Africa	M	−29.85	30.72	SE
*Rsi*	DM4739	Durban Natural Science Museum, Durban, South Africa	M	−30.42	30.68	SE
*Rsi*	DM5078	Durban Natural Science Museum, Durban, South Africa	M	−30.20	30.79	SE
*Rsi*	DM5442	Durban Natural Science Museum, Durban, South Africa	F	−30.27	30.59	SE
*Rsi*	DM6183	Durban Natural Science Museum, Durban, South Africa	M	−27.42	31.97	NE
*Rsi*	DM6890	Durban Natural Science Museum, Durban, South Africa	M	−27.42	31.97	NE
*Rsi*	DM7836	Durban Natural Science Museum, Durban, South Africa	F	−27.42	31.97	NE
*Rsw*	190612CHKRSW03	AES UCT, Cape Town, South Africa	F	−17.36	30.13	NZ
*Rsw*	260612MABRSW02	AES UCT, Cape Town, South Africa	M	−17.90	29.37	NZ
*Rsw*	260612MABRSW05	AES UCT, Cape Town, South Africa	F	−17.90	29.37	NZ
*Rsw*	120612LRSW01	AES UCT, Cape Town, South Africa	F	−18.91	32.69	NZ
*Rsw*	190612CHKRSW01	AES UCT, Cape Town, South Africa	M	−17.36	30.13	NZ
*Rsw*	200612DAMRSW04	AES UCT, Cape Town, South Africa	F	−16.83	31.23	NZ
*Rsw*	140612OGSRSW18	AES UCT, Cape Town, South Africa	F	−18.94	32.46	NZ
*Rsw*	140612OGSRSWIO	AES UCT, Cape Town, South Africa	F	−18.94	32.46	NZ
*Rsw*	220612MPCRSW02	AES UCT, Cape Town, South Africa	M	−16.09	29.46	NZ
*Rsw*	KM1760	Amathole Museum, King Williams Town, South Africa	F	−32.72	27.28	SE
*Rsw*	KM24302	Amathole Museum, King Williams Town, South Africa	M	−32.74	27.30	SE
*Rsw*	KM32610	Amathole Museum, King Williams Town, South Africa	F	−32.74	21.30	SE
*Rsw*	KM32611	Amathole Museum, King Williams Town, South Africa	F	−32.74	21.30	SE
*Rsw*	KM1762	Amathole Museum, King Williams Town, South Africa	M	−32.60	27.25	SE
*Rsw*	KM1763	Amathole Museum, King Williams Town, South Africa	F	−32.60	27.25	SE
*Rsw*	KM24286	Amathole Museum, King Williams Town, South Africa	F	−32.72	27.29	SE
*Rsw*	KM24287F	Amathole Museum, King Williams Town, South Africa	F	−32.72	27.29	SE
*Rsw*	KM24289F	Amathole Museum, King Williams Town, South Africa	M	−32.72	27.29	SE
*Rsw*	KM24291	Amathole Museum, King Williams Town, South Africa	F	−32.72	27.29	SE
*Rsw*	KM24296M	Amathole Museum, King Williams Town, South Africa	M	−32.72	27.29	SE
*Rsw*	KM24298	Amathole Museum, King Williams Town, South Africa	F	−32.74	21.30	SW
*Rsw*	KM24299	Amathole Museum, King Williams Town, South Africa	F	−32.74	21.30	SW
*Rsw*	KM24300	Amathole Museum, King Williams Town, South Africa	M	−32.74	21.30	SW
*Rsw*	KM24301M	Amathole Museum, King Williams Town, South Africa	M	−32.74	21.30	SW
*Rsw*	KM24303	Amathole Museum, King Williams Town, South Africa	F	−32.74	21.30	SW
*Rsw*	KM24304	Amathole Museum, King Williams Town, South Africa	F	−32.74	21.30	SW
*Rsw*	24293	Amathole Museum, King Williams Town, South Africa	M	−32.72	27.29	SE
*Rsw*	24292	Amathole Museum, King Williams Town, South Africa	F	−32.72	27.29	SE
*Rsw*	24288	Amathole Museum, King Williams Town, South Africa	M	−32.72	27.29	SE
*Rsw*	24295	Amathole Museum, King Williams Town, South Africa	M	−32.72	27.29	SE
*Rsw*	24290	Amathole Museum, King Williams Town, South Africa	M	−32.72	27.29	SE
*Rsw*	TM47159	Ditsong Museum, Pretoria, South Africa	F	−24.84	30.84	NE
*Rsw*	DM7080	Durban Natural Science Museum, Durban, South Africa	M	−29.93	29.77	NE
*Rsw*	mandibles922_86	Natural History Museum, Geneva, Switzerland	F	−9.96	25.97	DRC
*Rsw*	mandibles1046_47	Natural History Museum, Geneva, Switzerland	M	−11.00	26.71	DRC
*Rsw*	mandibles1046_49	Natural History Museum, Geneva, Switzerland	F	−11.00	26.71	DRC
*Rsw*	mandibles1046_57	Natural History Museum, Geneva, Switzerland	F	−10.41	27.55	DRC
*Rsw*	mandibles1046_63	Natural History Museum, Geneva, Switzerland	F	−11.00	26.59	DRC
*Rsw*	mandibles1047_52	Natural History Museum, Geneva, Switzerland	M	−10.41	27.55	DRC
*Rsw*	Skull922_86	Natural History Museum, Geneva, Switzerland	F	−9.96	25.97	DRC
*Rsw*	Skull1046_47	Natural History Museum, Geneva, Switzerland	M	−11.00	26.71	DRC
*Rsw*	Skull1046_63	Natural History Museum, Geneva, Switzerland	F	−11.00	26.59	DRC
*Rsw*	Skull1047_49	Natural History Museum, Geneva, Switzerland	F	−11.00	26.71	DRC
*Rsw*	Skull1047_52	Natural History Museum, Geneva, Switzerland	M	−10.41	27.55	DRC
*Rsw*	Skull1047_57	Natural History Museum, Geneva, Switzerland	F	−10.41	27.55	DRC

Abbreviation: *Rsi = Rhinolophus simulator*, *Rsw = Rhinolophus swinnyi*, F = female, M = male, Lat = latitude, Long = longitude, AES UCT = Animal Evolution and Systematics Lab at University of Cape Town; these skulls were extracted from voucher specimen captured during field work (2003–2013).

**TABLE A2:** 3D Landmark coordinates used for the skulls and mandibles of *Rhinolophus simulator* and *R. cf. simulator*

***Cranium***

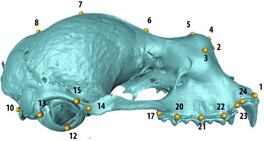

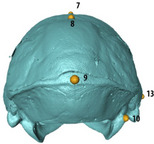

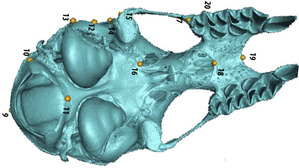

***Mandible***

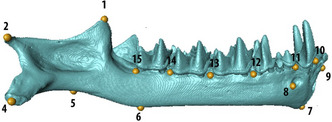

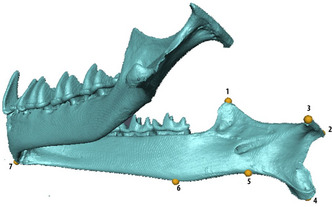

***Descriptions of the Landmarks shown in the diagrams***
***Cranium***
Most anterior point at the base of the canine Most anterior point of the anterior medial swelling on the midline between the left and right anterior medial swellings The widest point of the anterior medial swelling Most dorsal point of the anterior medial swelling Most posterior point of the anterior medial swelling on the midline of the skull Most anterior point of the sagittal crest on the midline of the skull Most dorsal point of the sagittal crest Most posterior and lowest point of the sagittal crest above the parietal depression Most posterior point of the skull at the sagittal and lambdoid crests Most lateral point of the occipital condyle Most Anterior point of the foramen magnum Most ventral point of the auditory bulla Most posterior point of the external auditory meatus at the junction with the paraoccipital process Widest point of the cranium where the zygomatic arch originates from the squamosal Widest point of the zygomatic arch Most posterior point of the foramen ovale End of tooth row at the base of the third molar Suture between the palatines at the midline Suture between the premaxilla and maxilla at the midline Between third and second molar at the base, facial side Between second and first molar at the base, facial side Between first molar and first premolar at the base, facial side Between first and second premolar at the base, facial side Between canine and first premolar at the base, facial side
***Mandible***
Tip of the coronoid process Tip of the condylar process at external edge Tip of the condylar process at internal edge Tip of the angular process midway along the width Point of extreme curvature at the incisura praemasseterica Point of extreme curvature on the lower edge of mandibular corpus Most anterior point of the lower edge of mandible corpus at the point where the two mandibles join Midpoint of the aboral edge of the mental foramen Most anterior point of the mandible corpus Midpoint at the base, between Incisor 1 and Canine 1, facial side Midpoint at the base, between C1 and PM1, facial side Midpoint at the base, between PM3 and M1, facial side Midpoint at the base, between M1 and M2, facial side Midpoint at the base, between M2 and M3, facial side At the end of Molar 3 (M3), on the facial side
